# Only the Carrot, Not the Stick: Incorporating Trust into the Enforcement of Regulation

**DOI:** 10.1371/journal.pone.0117212

**Published:** 2015-02-23

**Authors:** Juan P. Mendoza, Jacco L. Wielhouwer

**Affiliations:** 1 Accounting Department, VU University Amsterdam, Amsterdam, the Netherlands; 2 Accounting Department, VU University Amsterdam, Amsterdam, the Netherlands; Peking University, CHINA

## Abstract

New enforcement strategies allow agents to gain the regulator’s trust and consequently face a lower audit probability. Prior research suggests that, in order to prevent lower compliance, a reduction in the audit probability (the “carrot”) must be compensated with the introduction of a higher penalty for non-compliance (the “stick”). However, such carrot-and-stick strategies reflect neither the concept of trust nor the strategies observed in practice. In response to this, we define *trust-based regulation* as a strategy that incorporates rules that allow trust to develop, and using a generic (non-cooperative) game of tax compliance, we examine whether trust-based regulation is feasible (i.e., whether, in equilibrium, a reduction in the audit probability, without ever increasing the penalty for non-compliance, does not lead to reduced compliance). The model shows that trust-based regulation is feasible when the agent sufficiently values the future. In line with the concept of trust, this strategy is feasible when the regulator is uncertain about the agent’s intentions. Moreover, the model shows that (i) introducing higher penalties makes trust-based regulation less feasible, and (ii) combining trust and forgiveness can lead to a lower audit probability for both trusted and distrusted agents. Policy recommendations often point toward increasing deterrence. This model shows that the opposite can be optimal.

## Introduction

In this paper, we examine whether it is feasible to incorporate trust into an enforcement strategy. Our motivation is based on the following two observations. First, across diverse countries and regulatory settings, new enforcement strategies explicitly incorporate the concept of trust. In this context, trust is indicated by a reduction in the audit probability so that, as compared to other agents, trusted agents are audited less frequently. Second, prior research suggests that introducing a lower audit probability is indeed a feasible enforcement strategy [1–4], but only when agents face a relatively harsher punishment for not complying, which contradicts the concept trust [5].

Hence, the key question for both researchers and policy-makers is whether a strategy that reflects trust will inevitably lead to reduced compliance. In response to this, we define *trust-based regulation* as a strategy that incorporates rules that allow trust to develop (i.e., rules that determine how the audit probability can be reduced without ever increasing the penalty for not complying). Then, using a generic repeated game theoretical model of tax compliance, we examine whether trust-based regulation is a *feasible* strategy for the regulator (i.e., whether, in equilibrium, a lower audit probability does not lead to reduced compliance).

### References to trust in regulatory practice

In the area of tax regulation, at least 24 countries have recently established “trust-based relationships” between large corporate taxpayers and tax revenue bodies [6]. Similarly, the United Kingdom and the European Union explicitly incorporate trust into environmental regulations by promoting openness and cooperation between firms and supervisory authorities [4]. Recently, the Dutch Customs implemented a certification system in which auditing levels are based on the extent to which firms are trusted [7]. According to the Danish Code for Good Business Regulations, “businesses which comply with law should be rewarded with less supervision” [8]. For instance, firms in the food industry that pass four consecutive inspections will be subject to fewer inspections.

### Prior research on the reduction of the audit probability

Becker’s [9] economic model of criminal behavior shows that compliance increases with the penalty and the audit probability. The rationale is that more auditing and higher penalties increase the *expected costs* associated with not complying, and thus act as economic deterrents of non-compliance. Moreover, there exists an inverse relationship between audit probability and penalty, so that a reduction in one “compensates” an increase in the other. Through this “compensation” mechanism, the overall level of deterrence (i.e., the net expected costs associated with non-compliance) can be maintained.

The inverse relationship between audit probability and penalty is found in Becker’s model [9], as well as other classic one-period games of regulatory compliance [10–12]. This rationale applies to repeated games if we interpret these 1-period games as subgames in an infinitely repeated game (in this paper, we call this the “game without trust”). In such case, the solution of the 1-period game is equivalent to the solution of the repeated game. But even though repeated interactions may affect the players’ strategies (see, e.g., [13]), the same “compensation” logic is observed in multi-period games in which the agent’s expected costs depend on (i) the probability of detection, and (ii) any loss associated with getting caught not complying (imposed by the regulator). This can be seen, for example, in the repeated games of Greenberg and Harrington [1, 2]. The key point is that a lower audit probability leads to reduced compliance, *ceteris paribus*.

Models of conditional audit probabilities are particularly relevant in this context, as they have multiple periods and specifically examine the relationship between compliance and varying audit probabilities. In these models, the regulator uses the outcome of a current audit in order to determine an agent’s future audit probability [14]. Compliance can be rewarded with of a lower audit probability (the “carrot”), whereas non-compliance can be punished with a higher audit probability or penalty for non-compliance (the “stick”) [3]. In general, the “stick” refers to any action the regulator takes in order to *increase* the expected costs associated with non-compliance. In Greenberg’s model [1], the stick consists in entering an absorbing state with an audit probability equal to 1, while in Harrington’s model [2] the stick consists in entering a state with a higher audit probability and penalty for non-compliance. Subsequent refinements of these models incorporate this type of stick [3, 15]. The basic idea in these models is that the carrot needs to be “compensated” with a stick or else compliance decreases.

Conceptual work on reducing the audit probability builds on the same logic. According to a literature review on the topic, the introduction of a lower audit probability requires placing trust “in the foreground” and “distrust in the background” [16], i.e., the regulator’s response to trust violations should be incremental, and involve harsher penalties or “more punitive enforcement” [4]. For instance, the punishment of trust violations should be more severe as tax authorities increase their trust towards taxpayers [17].

### Trust-based regulation

A commonly applied definition of trust is the willingness to accept vulnerability based on positive expectations about the other’s behavior [5]. In line with this definition, trust has three additional characteristics. First, trust is a unidirectional construct, which means that it goes from a given trustor to a given trustee [18] (because this paper focuses on enforcement strategies, we only examine the regulator’s trust towards the agent, and ignore the agent’s trust towards the regulator). Second, trust can emerge as the relationship develops, which means that trust can be built, maintained, and dissolved [5, 19–21]. Third, trust involves risk as it makes the trustor vulnerable to opportunistic behavior. This implies that trust involves at least some level of uncertainty about the other’s actions [5].

In a regulatory context, a lower audit probability increases the expected benefits associated with not complying [9], and thus makes the regulator more vulnerable to non-compliance. Therefore, trust in this context can be defined as the regulator’s willingness to reduce the audit probability based on positive expectations about compliance. More specifically, trust is based upon the expectation that the agent will not comply less when the audit probability is lower.

Note that introducing the stick of a higher penalty is not indicative of the regulator’s trust. This stick deters the agent from not complying, and thus reduces the regulator’s vulnerability towards the agent. Moreover, this stick responds to negative rather than positive expectations about compliance. By increasing the penalty, the regulator expects the agent to take advantage of the lower audit probability and comply to a lesser extent. Hence, the stick reflects distrust rather than trust [5].

In sum, it can be said that reducing the audit probability while simultaneously increasing deterrence is “not trust at all” [5]. By definition, a strategy that incorporates trust cannot rely on making the punishment more severe. With this in mind, trust-based regulation can be defined as an enforcement strategy that incorporates rules that allow trust to develop, based on the introduction of the carrot of a lower audit probability, but never the stick of a higher penalty for not complying. This definition has the advantage of reflecting strategies that are currently applied in practice, and goes in line with the above-mentioned conceptualization of trust.

### Game overview

Our departure point is a generic model of tax compliance, which is repeated throughout an infinite time horizon (we refer to it as the “game without trust”). We present two rules that describe how trust is established, and then compare the game with and without these rules. As trust is more appropriately examined in settings that allow for opportunism, the model is a non-cooperative game in which players maximize their payoffs in an entirely selfish manner (we do not incorporate other-regarding preferences or reciprocity).

The generic model resembles the standard tax compliance model presented by Andreoni and colleagues [11]. Like influential models of tax compliance [22, 23], the generic model is based on economic deterrence, so that a lower audit probability reduces the incentives to comply, *ceteris paribus*. Focusing on a generic model of tax compliance allows us to directly analyze the consequences of trust-based regulation, while avoiding any confusion that may arise from incorporating additional extensions. Extensions to the generic model include, but are not restricted to, connections to the labor market, firm production, and social interaction among heterogeneous taxpayers [24]; information acquisition [25]; different orders of risk aversion [26]; and agency costs in corporate tax compliance [27]. For overviews of these and other extensions, see [11, 24, 28].

Moreover, the generic model captures the idea that (i) regulation involves repeated interactions between the agent and the regulator, (ii) compliance is costly for the agent, and (iii) auditing is costly for both the agent and the regulator. In this sense, the intuition of the game may apply to other regulatory settings with similar characteristics.

The generic model (i.e., the “game without trust”) represents the status quo; a setting in which trust cannot develop, and the regulator applies a standard “deterrence-based” approach. The penalty is the same in the games with and without trust. In the game with trust, however, the audit probability can be reduced. The implication is that trust can only benefit—and will never harm—the agent. The worst-case scenario for the agent is the status quo (i.e., a setting in which trust is not built or dissolves), and for this reason, incorporating the rules that allow trust to develop reflects a positive disposition towards the agent, which is a central aspect of trust [5].

The model shows that trust-based regulation is a feasible equilibrium strategy when the agent sufficiently values the future. If the agent’s time horizon is long, rewarding observed compliance with the carrot of a lower audit probability does not lead to reduced compliance in subsequent periods. This equilibrium is Pareto efficient as it never harms the regulator and reduces the agent’s costs of being audited.

However, if the agent’s time horizon is short, trust is misused, and therefore trust-based regulation is not an equilibrium strategy. Additionally, we show that trust-based regulation is feasible when (i) the regulator is uncertain about the agent’s intentions, and (ii) trust violations can be forgiven.

The Folk Theorem or grim strategies indicate that, for certain types of rules, these kind of equilibria exist [13, 29]. We confine our attention to the specific rules that are currently applied in practice and are indicative of the regulator’s trust towards the agent (i.e., a set of rules in which observed compliance leads to a lower audit probability without ever increasing penalties or audit probabilities). Of course, higher compliance can be achieved, for example, by increasing the penalties (as grim strategies suggest), but our objective is not to find the rules that lead to optimal compliance. Instead, we are interested in examining whether currently applied rules do not lead to trust violations.

Additionally, we confine our attention to the strategic agent, which may represent a firm or an individual and complies as long as it is profitable to do so. We do not analyze the honest agent, who complies regardless of the incentive to cheat [30]. This is because the main questions and risks faced in practice relate to opportunistic behavior. Nevertheless, in the Section *Equilibrium strategies* we briefly discuss how honesty makes trust-based regulation more beneficial for both players.

### Trust and reputation

Trust and reputation are different, yet closely related concepts [31]. In this subsection, we discuss the relation between these two concepts, and explain why the strategies applied in practice and modeled in our game resemble more a “trust-based” than a “reputation-based” strategy.

In game theory, reputation can be defined as the inference of a player’s private information based on past observed behavior [32]. Past behavior may refer to actions taken in an independent market [33], or more generally, in the same game but with other players [34, 35]. Once established, reputation may lead to what economists call “reputation effects”, which may include—but are not limited to—actions of reciprocity [36], trust [37], and fairness [38].

In line with the definition that we employ [5], trust in game theory can be defined as a belief about the other’s behavior that creates “the possibility of mutual benefit, if the other person is cooperative, and the risk of loss to oneself if the other person defects” [39]. Note, however, that it is possible to observe trust in the absence of reputation [40]. Indeed, one key distinction is that reputation can be an antecedent of trust (but not necessarily), and trust can in turn be an antecedent of a diversity of subsequent behaviors [41].

In our model, the audit probability is determined by behavior observed in the previous period, and the regulator always knows whether the agent is trusted or distrusted. In this sense, the model may resemble a “reputation-based” strategy. However, there are two reasons why “reputation-based” may not be the appropriate term to describe the regulator’s strategy. First, when the regulator has incomplete information about the agent’s discount factor, the initial strategy depends solely on expectations, and does not incorporate information from previous interactions (as these do not exist at this point). Second, past behavior is not observed in a different game or from previous interactions with different players; it is relevant *within* the relationship itself. Trust is derived from repeated interactions over time between the trustor and the trustee [5, 21, 42], while reputation is often referred to as information available prior to entering a relationship [31].

With this in mind, we suggest that the regulator’s strategy may be more appropriately described as “trust-based”. The model captures the key characteristics of strategies known as “trust-based” in practice, so at least it already approximates a currently applied conceptualization of trust. Additionally, from a lay perspective, lower monitoring is intuitively seen as an indicator of trust, whereas higher monitoring is seen as an indicator of distrust. Moreover, if we take compliance and enforcement games as a point of reference, the regulator’s vulnerability increases when he (i) reduces the audit probability without making the penalty more severe, and (ii) grants a trusted agent the benefit of the doubt. At least with respect to the standard theory in this area, this increased vulnerability is reflective of higher trust [5]. In expectation, the regulator is not worse off (indeed, trust is not necessarily irrational [43]), but this does not imply that he is not vulnerable to non-compliance.

The paper is organized as follows. The next sections contain the game description and equilibrium strategies. Then we analyze the consequences of incorporating uncertainty about the agent’s intentions, and forgiveness of trust violations. We then discuss the implications of our results, and present our conclusions.

## Game description

### The base model

The game has two players, an agent (*A*) and a regulator (*R*). The agent can either comply or not comply with the regulation, while the regulator can either audit the agent or not. The agent is assumed to choose the level of compliance that maximizes her expected payoffs. She complies with probability *α* and does not comply with probability 1 − *α*. Without loss of generality, the agent’s taxable income is assumed to be equal to 1. If the agent complies, she transfers a fraction of her income *τ* to the regulator, where *τ* ∈ (0, 1) represents the tax rate. An agent who is detected not complying is assumed to pay a penalty, *z*. When audited, the agent faces costs *c*.

Given that taxable income is normalized to 1, costs *c* are assumed to be between 0 and 1 − *τ*. Higher costs would mean that the payoff for the agent is negative when she is audited and complies. These costs may include, for instance, hiring professionals to attend the regulator’s inspection, or spending time to prepare and attend the audit [11].

As for the regulator, the cost of conducting an audit is equal to *s*, *s* ≥ 0. The regulator conducts an audit with probability *ρ* and no audit with probability 1 − *ρ*. The regulator is assumed to maximize its payoffs and therefore does not conduct an audit if the audit costs are larger than the benefits of detecting an agent not complying, so we assume that *s* ≤ *τ* + *z*. Otherwise, auditing does not represent a credible threat. We also assume that an audit allows the regulator to observe whether the agent pays the tax.

Note that in this model we ignore the possibility that the tax authority may retrieve information regarding compliance from the tax report. Adding this possibility increases complexity, but the qualitative results continue to hold when the level of reported income yields a signal about compliance to the tax authority, as long as the authority cannot infer full compliance with certainty from a reported income level. The model can also be interpreted as deciding whether or not to investigate compliance with respect to a specific tax reporting issue within the audit of a firm (e.g., transfer pricing). In this case, the tax report that reflects total taxable income is unlikely to provide a credible signal on the specific tax reporting issue.

The game is repeated at discrete points in time throughout an infinite time horizon. The agent and the regulator discount future payoffs by factors *δ*_*A*_ and *δ*_*R*_, respectively, with *δ*_*A*_, *δ*_*R*_ ∈ [0, 1] (for an example of a game in which players have different discount factors, see [44]).

The strategic form of the game, containing the payoffs for both players per time period, is presented in Table 1. Note that this is not a zero-sum game because both players face costs associated with supervision.

**Table 1:** Actions and payoffs per time period.

	**Comply (*α*)**		**Not comply (1 − *α*)**	
	***Payoff R***	***Payoff A***	***Payoff R***	***Payoff A***
**Audit (*ρ*)**	***τ* − *s***	**1 − *τ* − *c***	***τ* − *s* + *z***	**1 − *τ* − *c* − *z***
**Not audit** (1 − *ρ*)	***τ***	**1 − *τ***	**0**	**1**

Variables: *τ*: tax, *s*: costs of auditing, *c*: costs of being audited, *z*: penalty for not complying.

### Rules that allow trust to develop

The regulator establishes two rules concerning the ways in which trust can be built and dissolved. It is assumed that the agent is informed about these rules before the game starts. Throughout the game, the agent can be trusted, distrusted, or *waiting* to be either trusted or distrusted. A waiting agent remains waiting until the regulator conducts an audit. Once an agent is either trusted or distrusted, she will never be waiting again. The agent is neither trusted nor distrusted at the beginning of the game, and therefore the agent is assumed to be waiting in period 0.

**Rule 1: Building trust and the consequences of being trusted** A waiting agent becomes trusted if she is audited for the first time and the regulator observes that she is complying. That is, if the regulator detects a waiting agent complying in period *i*, then the agent will be trusted in period *i* + 1. Trust has two observable consequences.

The first consequence is that, as compared to a distrusted agent, a trusted agent faces a lower audit probability. More specifically, for any period *i* ≥ 1, a trusted agent faces an audit probability, *ρ*_*t*, *i*_, which is lower than the audit probability faced by a distrusted agent, *ρ*_*d*, *i*_, where the subscripts *t* and *d* stand for “trusted” and “distrusted”, respectively. The difference between *ρ*_*d*, *i*_ and *ρ*_*t*, *i*_ reflects the regulator’s increased level of vulnerability, and may thus be interpreted as an indicator of trust [45]. If it turns out that in equilibrium *ρ*_*t*, *i*_ > *ρ*_*d*, *i*_, we conclude that trust-based regulation is not feasible because assigning a *higher* audit probability to a trusted agent contradicts the concept of trust [5].

The second consequence is that a trusted agent is given the benefit of the doubt. This means that a trusted agent remains trusted when no audit is conducted, just as if compliance had been confirmed. The two consequences imply that trust is maintained (i.e., the agent continues facing a lower audit probability) when (i) the regulator audits the trusted agent and the agent is indeed complying, or (ii) the regulator does not audit the agent. Note that, in the absence of an audit, a trusted agent can misuse trust by not complying, and still remain being trusted. Overall, this rule implies that the regulator enters a state of increased vulnerability with a trusted agent. Moreover, this rule approximates the idea that trust is built depending on what is observed through experience and interaction [21, 42].

**Rule 2: Dissolving trust and the consequence of being distrusted** A waiting or trusted agent will become distrusted if the regulator conducts an audit and the agent is not complying. In other words, an agent caught not complying in period *i* will be distrusted in period *i+1*.

The consequence of being distrusted is that the agent will remain distrusted from then on (i.e., the distrusted state is an absorbing state), and thus loses the benefit of facing a lower audit probability as in the trusted state. This rule captures the idea that a trustor is unwilling to become vulnerable when the trustee’s behavior is evidently negative. To illustrate this point, consider the following principle followed by the tax authorities in the Netherlands: “we trust our taxpayer unless we have information they cannot be trusted” [46]. We do not incorporate forgiveness at this point in the game. In the Section *Unknown discount factor and forgiveness* we elaborate on the consequences of forgiveness.

**Fig 1:**
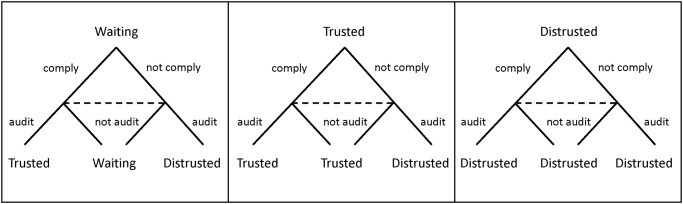
The agent’s possible states.

Fig. 1 presents the agent’s possible states. The state-dependent strategies of the agent in period *i* are denoted *α*_*k*, *i*_ for state *k* ∈ {*w*, *t*, *d*}, i.e., when the agent is waiting, trusted, or distrusted, respectively. Similarly, the state-dependent strategies of the regulator are denoted *ρ*_*k*, *i*_ for state *k* ∈ {*w*, *t*, *d*}. For notational convenience, however, the dependence on *i* will often be suppressed because the strategies are the same for different time periods.

### Values and payoffs

In period *i*, the agent’s value of the game in state *k* from period *i* until infinity is denoted *VA*_*k*, *i*_, and for the regulator, *VR*_*k*, *i*_. Taking the above rules into account, we can now present the agent’s and regulator’s expected values for each of the agent’s possible states in period *i* (as explained before, we suppress the subscript *i*).

The agent’s values are:

$$\begin{array}{c} \begin{array}{ccc} \text{Distrusted:} & VA_{d}= & 1-\alpha_{d}\tau -\rho_{d}\;\left(1-\alpha_{d}\right)\;\left(\tau +z\right)-\\ & & \rho_{d}c+\delta_{A}VA_{d}\\ \text{Trusted:} & VA_{t}= & 1-\alpha_{t}\tau -\rho_{t}\;\left(1-\alpha_{t}\right)\;\left(\tau +z+\delta_{A}\;\left(VA_{t}-VA_{d}\right)\right)-\\ & & \rho_{t}c+\delta_{A}VA_{t}\\ \text{Waiting:} & VA_{w}= & 1-\alpha_{w}\tau -\rho_{w}\;\left(1-\alpha_{w}\right)\;\left(\tau +z+\delta_{A}\;\left(VA_{t}-VA_{d}\right)\right)-\\ & & \rho_{w}\;\left(c+\delta_{A}\;\left(VA_{w}-VA_{t}\right)\right)+\delta_{A}VA_{w}. \end{array} \end{array}$$ (1)

The regulator’s values are:

$$\begin{array}{c} \begin{array}{ccc} \text{Distrusted:} & VR_{d}= & \alpha_{d}\tau +\rho_{d}\;\left(1-\alpha_{d}\right)\;\left(\tau +z\right)-\\ & & \rho_{d}s+\delta_{R}VR_{d}\\ \text{Trusted:} & VR_{t}= & \alpha_{t}\tau +\rho_{t}\;\left(1-\alpha_{t}\right)\;\left(\tau +z-\delta_{R}\;\left(VR_{t}-VR_{d}\right)\right)-\\ & & \rho_{t}s+\delta_{R}VR_{t}\\ \text{Waiting:} & VR_{w}= & \alpha_{w}\tau +\rho_{w}\;\left(1-\alpha_{w}\right)\;\left(\tau +z-\delta_{R}\;\left(VR_{t}-VR_{d}\right)\right)-\\ & & \rho_{w}\;\left(s-\delta_{R}\;\left(VR_{t}-VR_{w}\right)\right)+\delta_{R}VR_{w}, \end{array}\; \end{array}$$ (2)

where *α*_*k*_ is the agent’s compliance probability, and *ρ*_*k*_ is the regulator’s audit probability in state *k* ∈ {*w*, *t*, *d*}.

## Equilibrium strategies

The game is solved by making the regulator indifferent between auditing and not auditing, and the agent indifferent between complying and not complying. It can be verified from (1) that, for each state, the regulator makes the agent indifferent with the following strategies:

$$\begin{array}{ccc} \rho_{d} & = & \frac{\tau }{\tau +z} \end{array}$$ (3)

$$\begin{array}{ccc} \rho_{t} & = & \frac{\tau }{\tau +z+\delta_{A}\;\left(VA_{t}-VA_{d}\right)} \end{array}$$ (4)

$$\begin{array}{ccc} \rho_{w} & = & \rho_{t}. \end{array}$$ (5)

For the description of how these strategies are derived, see the proof of Proposition 1 in S1 Appendix.

The intuition is that an agent in the trusted state bears the risk of losing her advantage (i.e., a reduction in expected audit costs). When the agent values trust (i.e., when *VA*_*t*_ > *VA*_*d*_), the regulator can lower the audit probability. As the benefits of trust increase, non-compliance is less attractive, which results in the regulator’s preference for a lower audit probability. Note that when being trusted is not valuable (i.e., when *VA*_*t*_ = *VA*_*d*_), the audit probability is the same in all states and equal to *ρ*_*d*_. When the agent does not value trust (i.e., *VA*_*t*_ < *VA*_*d*_), the audit probability in the trust state is *higher* than in the distrusted state, which violates the principle of trust. Furthermore, it is immediately seen that the regulator strategically starts the game by applying an audit probability *ρ*_*w*_ equal to the audit probability assigned to a trusted agent, *ρ*_*t*_. In other words, it is optimal for the regulator to start the game with trust. This is possible because the agent faces the risk of becoming distrusted forever, as she remains in the waiting state until the first audit takes place.

Similarly, the regulator is indifferent between auditing and not auditing when

$$\begin{array}{ccc} \alpha_{d} & = & \frac{\tau +z-s}{\tau +z}=1-\frac{s}{\tau +z} \end{array}$$ (6)

$$\begin{array}{ccc} \alpha_{t} & = & \frac{\tau +z-s-\delta_{R}\;\left(VR_{t}-VR_{d}\right)}{\tau +z-\delta_{R}\;\left(VR_{t}-VR_{d}\right)} \end{array}$$ (7)

$$\begin{array}{ccc} \alpha_{w} & = & \frac{\tau +z-s-\delta_{R}\;\left(VR_{w}-VR_{d}\right)}{\tau +z-\delta_{R}\;\left(VR_{t}-VR_{d}\right)} \end{array}$$ (8)

Note that the value functions depend on the audit and compliance probabilities, which yields a system of equations that can be solved to find the optimal strategies in terms of the model’s parameters. The resulting equilibrium strategies and game values are given in the following proposition.

**Proposition 1** *The following strategies constitute the subgame perfect equilibria for each period*.

- *Traditional (purely) deterrence-based solution*
  $$\begin{array}{c} \begin{array}{cccc} \rho_{k}=\frac{\tau }{\tau +z} & & VR_{k}=\frac{\alpha_{k}\tau }{1-\delta_{R}} & \forall \;k\in \{w,\;d,\;t\}\\ \alpha_{k}=1-\frac{s}{\tau +z} & & VA_{k}=\frac{1-\tau -\rho_{k}c}{1-\delta_{A}} & \forall \;k\in \{w,\;d,\;t\} \end{array} \end{array}$$
- *Trust-based solution*
  $$\begin{array}{c} \begin{array}{cccc} \left\{\begin{array}{cc} \rho_{d} & =\frac{\tau }{\tau +z}\\ \rho_{t}=\rho_{w} & =\frac{\tau +z}{c}\frac{1-\delta_{A}}{\delta_{A}} \end{array}\right. & & VR_{k}=\frac{\alpha_{k}\tau }{1-\delta_{R}} & \forall \;k\in \{w,\;d,\;t\}\\ \alpha_{k}=1-\frac{s}{\tau +z} & & VA_{k}=\frac{1-\tau -\rho_{k}c}{1-\delta_{A}} & \forall \;k\in \{w,\;d,\;t\} \end{array} \end{array}$$

*The trust-based solution satisfies ρ_*d*_ > ρ_t_ iff* $\delta_{A}>\delta^{⋆}=\frac{\tau +z}{\tau +z+c\rho_{d}}$.

The proof can be found in S1 Appendix. The first solution corresponds to the traditional (purely) deterrence-based solution, in which the agent is treated equally in all states. Without trust-based regulation, the game solution is a mixed-strategy in which the strategic agent complies with probability $1-\frac{s}{\tau +z}$, and the regulator audits with probability $\frac{\tau }{\tau +z}$. The compliance probability decreases with the regulator’s supervision costs *s*, and increases with the benefits of detecting non-compliance, *τ* + *z*. There is an inverse relationship between the audit probability and the penalty *z*, which is the basic principle of deterrence [9]. Because both players are made indifferent, the regulator’s expected payoff in any period *i* equals *τα*_*i*_ = *τ* − *sρ*_*i*_, whereas the agent’s expected payoff equals 1 − *τ* − *cρ*_*i*_.

Trust-based regulation yields a second equilibrium, referred to as the *trust-based solution*. It shows that, when the agent sufficiently values the future (i.e., when *δ*_*A*_ > *δ* ^⋆^), there exists an equilibrium in which the agent enters the trust state based on observed compliance, the audit probability in the trusted state is lower than in the distrusted state (i.e., *ρ*_*t*_ < *ρ*_*d*_), and the compliance probability in both states is the same. This means that, when *δ*_*A*_ > *δ* ^⋆^, the regulator’s trust is *not* misused. The intuition is that this compliance level makes the regulator indifferent between auditing and not auditing so that different audit probabilities result in the same payoff. When *δ*_*A*_ < *δ* ^⋆^, the trust-based solution would result in a higher audit probability in the trust state (i.e, *ρ*_*t*_ > *ρ*_*d*_), as otherwise the regulator’s trust would be misused. In such case, the regulator’s strategy would not reflect the principle of trust. Moreover, this strategy would increase costs without benefits for either the agent or the regulator. Hence, trust-based regulation is not incorporated when *δ*_*A*_ < *δ* ^⋆^.

The probability of auditing a trusted agent, *ρ*_*t*_, depends on the extent to which the agent values future payoffs. More precisely, as *δ*_*A*_ becomes higher, *ρ*_*t*_ can be lower because the future benefits of being trusted are more valuable to the agent. This implies that there is an appropriate level of trust for each agent, so that the level of trust *ρ*_*d*_ − *ρ*_*t*_ increases with each agent’s *δ*_*A*_.

Being trusted yields an advantage. The audit-related costs for the agent become less as the expected interaction with the regulator decreases. When these costs (*c*) approach zero, the trust-based solution with *ρ*_*t*_ < *ρ*_*d*_ is not sustainable, which follows from the fact that *δ* ^⋆^ would be equal to one.

Interestingly, the solution shows that increasing deterrence undermines the feasibility of trust-based regulation. This can be seen from three different angles. First, the relationship between audit probability and penalty is opposite in the trusted and distrusted states. Whereas *ρ*_*d*_ is decreasing in *z*, *ρ*_*t*_ is increasing in *z*. This means that the introduction of a higher penalty, which resembles a purely deterrence-based approach, requires a higher (rather than lower) audit probability in the trusted state. Second, the difference between audit probabilities in the trusted and distrusted states decreases when *z* becomes higher. Third, if we substitute *ρ*_*d*_ in *δ* ^⋆^, it can be seen that *δ* ^⋆^ is increasing in *z*, and as *δ* ^⋆^ becomes higher, trust-based regulation becomes feasible for fewer values of *δ*_*A*_. The intuition is that, by increasing the penalty, the regulator reduces the agent’s benefits of being trusted, and therefore the regulator prefers to increase the audit probability in the trusted state.

Now let us consider the effects on social costs. The trust-based solution yields no benefits to the regulator in terms of total payoffs. The reduced audit costs are offset by fewer taxes and penalties received. The regulator is made indifferent between auditing and not auditing and can thus vary its audit probability without affecting its expected value. However, there is a benefit for the agent. The value in the trusted state is strictly higher than the value in the distrusted state because the expected costs of being audited are lower. Given that taxes and penalties are transfers between the players, social costs are equal to expected costs associated with auditing (for the regulator) and being audited (for the agent), *ρ*_*i*_(*s* + *c*). As long as the agent enters the trusted state, social costs decrease by (*ρ*_*d*_ − *ρ*_*t*_)(*s* + *c*) for each period in which the agent is trusted. However, one should note that under the current rules and solution, these benefits disappear in the long run, as soon as the agent enters the absorbing state of being distrusted. In order to prevent the agent from becoming distrusted forever, the regulator can incorporate forgiveness. We will elaborate on the effects of forgiveness in the next section.

Finally, let us intuitively elaborate on the effects of incorporating honesty into this model. In a setting with multiple agents, if a fraction of agents is honest and always complies (as in [30]), these agents will always remain in the trusted state, and then the distrusted state is no longer an absorbing state. Honest agents are never caught not complying, and *ρ*_*w*_ = *ρ*_*t*_ implies that all honest agents are initially trusted. In such case, a trust-based solution rewards and safeguards honesty, reduces audit costs, and ultimately benefits both agents and the regulator. With honesty, this solution is always preferred over the traditional (purely) deterrence-based solution.

## Unknown discount factor and forgiveness

### Unknown discount factor *δ*_*A*_

The previous section shows that the amount of increased trust depends on the agent’s intentions, which are reflected by *δ*_*A*_. Incorporating the regulator’s uncertainty about *δ*_*A*_ is relevant because this is likely to be the case in practice, and moreover, this more explicitly captures the notion of trust, as trust is based on expectations rather than complete certainty about the other’s intentions [5].

In order to incorporate uncertainty, let us first assume that the agent’s discount factor is drawn from some distribution function that is common knowledge, the realization of the agent’s discount factor is not observed by the regulator, and the regulator learns the agent’s discount factor after the first audit.

In line with the concept of trust, the following proposition shows that trust-based regulation is feasible when the regulator has positive expectations about the agent’s intentions (i.e., when there is a strictly positive probability that the agent will not violate the regulator’s trust), and if these expectations are sufficiently high, trust may be initially granted (in the waiting state). Let us first define *α* ^⋆^ as the compliance probability obtained in Proposition 1, $1-\frac{s}{\tau +z}$.

**Proposition 2** *When δ_A_ is unknown to the regulator, trust-based regulation is a feasible strategy (ρ_t_ ≤ ρ_d_) as long as* Pr{δ_A_ > δ^⋆^} > 0. *Moreover*,

- *when positive expectations are sufficiently high, trust is initially granted, and compliance is higher than in the game without trust, i.e., if* Pr{δ_*A*_ > δ^⋆^} > α^⋆^, *then E*[*α_w_*] > *α* ^⋆^ *and ρ*_*w*_ < *ρ*_*d*_;
- *when positive expectations are not sufficiently high, the agent is initially distrusted, and compliance is not affected by trust-based regulation, i.e., if* Pr{*δ*_*A*_ > *δ* ^⋆^} ≤ *α* ^⋆^, *then E*[*α*_*w*_] = *α* ^⋆^ *and ρ*_*w*_ = *ρ*_*d*_.

The proof can be found in S1 Appendix. The intuition is as follows. The regulator starts the game by making an assessment of the probability that the agent has positive intentions, Pr{*δ*_*A*_ > *δ* ^⋆^}. Trust-based regulation is not a feasible strategy when the regulator knows that the agent intends to misuse trust, Pr{*δ*_*A*_ > *δ* ^⋆^} = 0. However, when there is even a small probability that the agent has positive intentions, trust-based regulation can be introduced without reducing the regulator’s expected payoffs.

Let us start with case *i* of Proposition 2. Given a *ρ*_*w*_ < *ρ*_*d*_, there exists a critical level of *δ*_*A*_, denoted $\tilde{\delta }$, such that the agent has a pure strategy to comply when $\delta_{A}>\tilde{\delta }$ (i.e., when the benefits of honoring trust outweigh the advantages of misusing trust), and a pure strategy not to comply when $\delta_{A}<\tilde{\delta }$ (i.e., when, in the short term, the agent benefits from a lower probability of getting caught not complying). Now, if the regulator were to apply *ρ*_*w*_ = *ρ*_*d*_ in the waiting state, expected compliance would, given the optimal response of complying whenever $\delta_{A}<\tilde{\delta }$, exceed *α* ^⋆^. It follows that the regulator optimally responds by lowering *ρ*_*w*_. The optimal solution cannot have the (even lower) *ρ*_*w*_ that would result in compliance equal to *α* ^⋆^, as it follows that the regulator then maximizes its value by increasing *ρ*_*w*_. This leads to *α*_*w*_ > *α* ^⋆^.

The resulting audit probability in the waiting state $\tilde{\rho }<\rho_{d}$ is equal to the audit probability of a trusted agent with discount factor $\tilde{\delta }$, and is decreasing in *δ*_*A*_. This implies that, if the regulator conducts an audit with probability $\rho_{w}=\tilde{\rho }$, and finds that the agent is complying, then the regulator under-estimated the agent’s discount factor (i.e., $\delta_{A}\geq \tilde{\delta }$). It can be concluded that the agent has the intention not to misuse trust, and can thus receive a lower audit probability in the trusted state (*ρ*_*t*_ ≤ *ρ*_*w*_). Increasing the level of trust, based on previous (positive) interactions, corresponds both to what is observed in practice and the idea that trust can develop through time [5].

When these positive expectations are not sufficiently high (case *ii*), the probability that the agent complies is less than *α* ^⋆^ for all *ρ*_*w*_ < *ρ*_*d*_, implying that the regulator can only start the game with *ρ*_*w*_ = *ρ*_*d*_. With this audit probability, an agent with *δ*_*A*_ < *δ* ^⋆^ is indifferent between complying and not complying, and an agent with *δ*_*A*_ > *δ* ^⋆^ will always prefer to comply. With the rules that allow trust to develop in place, complying with probability $\alpha_{w}=\frac{\alpha^{⋆}-\text{Pr}\left\{\delta_{A}>\delta^{⋆}\right\}}{\text{Pr}\left\{\delta_{A}<\delta^{⋆}\right\}}$ when *δ*_*A*_ < *δ* ^⋆^ implies that *ρ*_*w*_ = *ρ*_*d*_ is indeed the optimal strategy of the regulator. It thus follows that in case *ii*, granting initial trust cannot be an equilibrium strategy, although it is possible to build trust over time if compliance is later confirmed. This result also fits the idea that trust emerges from the relationship itself, and evolves based on what is learned through repeated interactions [5, 21].

To illustrate how trust can develop through time, consider the case of the tax and customs authorities in the Netherlands, who shape their supervision strategies based on what they learn from repeated interactions with each firm (these are also known as tailor-made supervision strategies, [7]).

### Forgiveness

Until this section, we have assumed that the distrusted state is an absorbing state. Considering that amnesty programs and similar instruments are used in practice, in this subsection we examine the effects of forgiving trust violations. When the regulator observes a distrusted agent complying, the agent returns to the waiting state with probability *u*, and remains in the distrusted state with probability 1 − *u* (as in [2]). Note that *u* = 0 corresponds to the earlier case with no forgiveness, and *u* = 1 implies immediate forgiveness. It can be verified from the formulation of the value functions below that for *u* = 1 only the traditional (purely) deterrence-based solution exists. This immediately implies that too much forgiveness will destroy the feasibility of trust-based regulation. The players’ values in the trusted and waiting states are the same as in (1) and (2). The values in the distrusted state are however different:

$$\begin{array}{c} \begin{array}{cc} VR_{d}= & \alpha_{d}\tau +\rho_{d}\;\left(1-\alpha_{d}\right)\;\left(\;\tau +z\right)-\\ & \rho_{d}\;\left(s-\alpha_{d}u\delta_{R}\;\left(\;VR_{t}-VR_{d}\right)\right)+\delta_{R}VR_{d}\\ VA_{d}= & 1-\alpha_{d}\tau -\rho_{d}\;\left(1-\alpha_{d}\right)\;\left(\tau +z\right)-\\ & \rho_{d}\;\left(c-\alpha_{d}u\delta_{A}\;\left(VA_{t}-VA_{d}\right)\right)+\delta_{A}VA_{d}. \end{array} \end{array}$$ (9)

Making the agent and the regulator indifferent in each of the states yields the following strategies in terms of value functions:

$$\begin{array}{c} \begin{array}{cccc} \rho_{d}= & \frac{\tau }{\tau +z+\delta_{A}u\left(VA_{w}-VA_{d}\right)} & \alpha_{d}= & \frac{\tau +z-s}{\tau +z-\delta_{R}u\left(VR_{w}-VR_{d}\right)}\\ \rho_{t}= & \frac{\tau }{\tau +z+\delta_{A}\left(VA_{t}-VA_{d}\right)} & \alpha_{t}= & \frac{\tau +z-s-\delta_{R}\left(VR_{t}-VR_{d}\right)}{\tau +z-\delta_{R}\left(VR_{t}-VR_{d}\right)}\\ \rho_{w} & =\rho_{t} & \alpha_{w}= & \frac{\tau +z-s-\delta_{R}\left(VR_{w}-VR_{d}\right)}{\tau +z-\delta_{R}\left(VR_{t}-VR_{d}\right)} \end{array} \end{array}$$ (10)

The solution to this system of equations yields the equilibrium strategies as given in the following proposition.

**Proposition 3** *For each period of the game with forgiveness, the following strategies constitute the subgame perfect equilibria*.

- *Traditional (purely) deterrence-based solution*
  $$\begin{array}{c} \begin{array}{ccc} \rho_{k} & =\frac{\tau }{\tau +z} & \text{for}\;\text{all}\;k\in \{w,d,t\}\\ \alpha_{k} & =1-\frac{s}{\tau +z} & \text{for}\;\text{all}\;k\in \{w,d,t\} \end{array} \end{array}$$
- *Trust-based solution*
  $$\begin{array}{c} \begin{array}{c} \left\{\begin{array}{cc} \rho_{t}=\rho_{w} & =\frac{1}{2c\delta_{A}\left(1-u\right)}\left[F+\sqrt{F^{2}+4c\delta_{A}\left(1-u\right)\tau u\left(1-\delta_{A}\right)}\right]\\ \rho_{d} & =\frac{\tau +\rho_{t}c}{\tau +z+c}+\frac{\left(1-\delta_{A}\right)\left[1-\rho_{t}\left(\tau +z\right)\right]}{\delta_{A}\rho_{t}\left(\tau +z+c\right)} \end{array}\right.\\ \alpha_{k}=1-\frac{s}{\tau +z}\;\text{for}\;\text{all}\;k\in \left\{w,\;d,\;t\right\} \end{array} \end{array}$$
  *where F* = *τuδ*_*A*_ + (*τ* + *z*)(1 − *δ*_*A*_)(1 − *u*).

*For δ*_*A*_ > *δ* ^⋆^, *as given in Proposition 1, there exists a u* ^⋆^ ∈ (0, 1) *so that the trust-based solution satisfies ρ*_*d*_ > *ρ*_*t*_ iff *u* < *u* ^⋆^, *where* $u^{⋆}=\frac{c\tau \delta_{A}-(1-\delta_{A}){\left(\tau +z\right)}^{2}}{\delta_{A}\tau (c+\tau +z)}$.

This proposition shows that when trust-based regulation is feasible (*δ*_*A*_ > *δ* ^⋆^), forgiveness can also be included, as long as the regulator is not *too* forgiving. This has an effect on the audit probabilities both in the trusted and distrusted states. The possibility of being forgiven and receiving the benefits of trust increases the value in the distrusted state, and for this reason, the regulator can even reduce the audit probability in the distrusted state, although this would be a modest reduction. So compared to the game without trust, the audit probability in this game is reduced in both the trusted and distrusted states without affecting compliance.

With forgiveness (*u* > 0), the distrusted state is no longer an absorbing state. Taking the trusted and waiting states together (which are treated equally by the regulator), and writing the transitions as a Markov chain shows that the probability that—in the long run—an agent is in the trusted state, Π_*t*_, is equal to

$$\begin{array}{c} \Pi_{t}=\frac{\rho_{d}u\alpha_{d}}{\rho_{d}u\alpha_{d}+\rho_{t}\;\left(1-\alpha_{d}\right)}>0. \end{array}$$

This implies that the reduced costs of auditing and being audited in the trusted state are realized not only in the short, but also in the long run.

Let us now illustrate this equilibrium and its long term effects with a numerical example.

**Fig 2:**
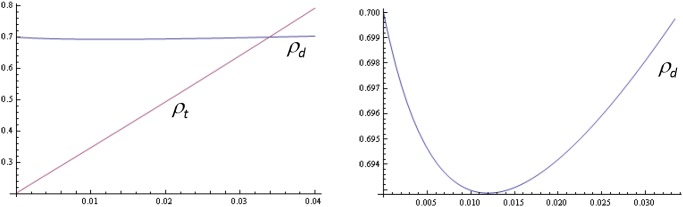
Numerical example. Optimal audit probabilities *ρ*_*d*_ and *ρ*_*t*_ as a function of forgiveness *u*, for *τ* = 0.35, *z* = 0.15, *δ* = 0.99 and *c* = 0.025. Panel A: *ρ*_*d*_, *ρ*_*t*_ as a function of *u*         Panel B: *ρ*_*d*_ on smaller scale

**Example 1** *Let τ* = 0.35, *z* = 0.15, *and c* = 0.025. *It follows from these parameter values that δ* ^⋆^ = 0.966. *Therefore, if δ*_*A*_ > 0.966, *trust-based regulation is a feasible solution in the absence of forgiveness. To illustrate the effect of forgiveness, let us assume that δ*_*A*_ = 0.99. Fig. 2 *presents the equilibrium audit strategies*.

*For these parameter values, the traditional deterrence-based solution has an audit probability equal to* $\frac{\tau }{\tau +z}=0.7$. *In the absence of forgiveness, the audit probability in the trusted state (ρ_t_) is* 0.2 *(see Proposition 1). Forgiveness (i.e., u* > 0) *makes ρ_t_ increase, and the trust-based solution is feasible with ρ_t_* < *ρ_d_ as long as forgiveness u is less than u* ^⋆^ = 0.0334. *In a setting with multiple agents, this implies that at most* 3.34% *of distrusted agents who are found complying can be forgiven. The minimum audit probability in the distrusted state is obtained with forgiveness u* = 0.012, *which yields ρ_d_* = 0.6929.

*Let the cost of auditing be s* = 0.25, *which yields α* = 0.5. *For this value and for u* = 0.012, Π_*t*_ = 0.0216, *which means that the percentage of trusted agents in the long run is* 2.16%. *The audit probabilities are ρ_t_* = 0.377 *and ρ_d_* = 0.693. *In the traditional solution, the expected social costs per period (i.e., the total costs of auditing and being audited) are equal to* 0.193, *and in the trust-based solution they are equal to* 0.189, *implying a cost reduction in the long run of* 2.00%. *In the short run, the cost reduction is* 53.80%.

*Let us finally consider the effect of improved efficiency. When the cost of auditing s is assumed to be only* 0.1, *compliance increases, and more agents end up being trusted in the long run* (Π_*t*_ = 0.0811), *which yields a social cost reduction of* 4.69% *in the long run*.

Two key points can be taken from this example. First, as expected, forgiveness reduces the amount of trust that trusted agents receive: the difference between *ρ*_*d*_ and *ρ*_*t*_ becomes smaller. Second, as compared to the traditional deterrence-based game, trust-based regulation with a low level of forgiveness allows the regulator to reduce the audit probability, independently of the state in which the agent is, without affecting compliance.

## Implications

These results have five important implications, which might be relevant to the policy-maker.

*Implication 1: Trust-based regulation can reduce social costs*. A feasible trust-based regulation strategy allows the regulator to reduce the audit probability in the trusted state, and with low levels of forgiveness, reduce the audit probability in *all* states (trusted and distrusted). Because audits are costly for both players, this strategy reduces social costs.

*Implication 2: Trust-based regulation is not always possible*. Some may promote the unconditional incorporation of trust into regulatory practice. However, we show that trust is misused when the cost of being audited is very small or when the agent is short-term oriented. The model shows that a naive strategy based on “blind trust” is not recommendable, as having positive expectations is required.

*Implication 3: The level of trust is determined by each individual relationship*. A reduction in the audit probability depends on the regulator’s expectations or actual knowledge about the agent’s discount factor. In practice, the regulator may decide the extent to which each agent is trusted, depending on what it learns about each agent. This reflects a system of “tailor-made” strategies, in which trust may develop on a selective case-by-case basis.

*Implication 4: Increasing deterrence undermines trust*. The model shows that higher penalties make trust-based regulation less feasible. This goes in line with the idea that trust and deterrence can not go hand in hand.

*Implication 5: Positive expectations suffice*. When the regulator is uncertain about the agent’s intentions, but nevertheless has positive expectations about her intentions, trust-based regulation does not reduce the regulator’s payoffs, and may even increase compliance.

Before concluding, let us mention that these results may apply to not only a tax setting but also other regulatory settings. Consider a general case in which the agent decides whether to pay a compliance cost *k*, yielding a benefit to the regulator equal to *b*. This benefit may be either political or driven by a direct economic transfer. The payoffs in each period for this model are given in Table 2.

**Table 2:** Actions and payoffs per time period for a general model.

	**Comply (*α*)**		**Not comply (1 − *α*)**	
	***Payoff R***	***Payoff A***	***Payoff R***	***Payoff A***
**Audit** (*ρ*)	***b* − *s***	**−*k* − *c***	***b* − *s* + *z***	**−*k* − *c* − *z***
**Not audit** (1 − *ρ*)	***b***	**−*k***	**0**	**0**

Variables: *b*: regulator’s benefit derived from compliance, *k*: cost of complying, *s*: costs of auditing, *c*: costs of being audited, *z*: penalty for not complying.

For this model, the optimal strategies are equal to the strategies derived in Propositions 1 and 3, with *τ* replaced by *b* in the strategy of *A*, and by *k* in the strategy of *R*. Note that each player’s strategy is a function of the other player’s parameters, as each strategy is derived to make the other player indifferent.

## Conclusion

In this paper, the regulator has the option to incorporate a trust-based strategy in which compliance is rewarded with a lower audit probability. Prior research indicates that reducing the audit probability (the carrot) must be compensated with the introduction of harsher penalties for non-compliance (the stick [1, 2]). The model in this paper shows that introducing the carrot without the stick is a feasible strategy when the agent sufficiently values the future.

By focusing on a non-cooperative game, we are able to examine whether a trust-based strategy is feasible even in the most severe case (i.e., without coalitions, cooperative commitments, or other-regarding preferences). The direct implication is that a feasible trust-based strategy will ultimately respond to self-interest (for both players).

A question that may arise is whether such strategy can respond to a selfish motive and at the same time approximate the concept of trust. To answer this, consider the following three possibilities. By introducing a new type of regulation, the regulator’s expected payoffs can decrease, increase, or remain the same. If trust-based regulation were to *reduce* the regulator’s payoffs, introducing this type of regulation would be—from an economic perspective—irrational, and the analysis would lie beyond the scope and theory of this paper. Conversely, if trust-based regulation were to *increase* the regulator’s payoffs, it would be more indicative of an exclusively profit-maximizing strategy. Only when the regulator is *indifferent* about introducing trust-based regulation, this strategy takes the furthest distance from a purely profit-maximizing strategy without violating the principle of rationality. Because trust-based regulation has the potential to only benefit the agent, without ever reducing the expected payoffs of any player, the regulator’s indifference best reflects a positive disposition towards the agent—as much as it is possible in a non-cooperative game.

More generally, it may be argued that this “trust-based” strategy does not capture the notion of trust but rather a system of incentives in which the regulator maximizes payoffs [47]. Note, however, that incorporating trust may be based on both calculative and non-calculative motives, so the calculativeness nature of a trust-based strategy does not necessarily rule out the existence of trust [43].

Another question is whether it is possible to introduce trust without a certain degree of reciprocity. Reciprocity can be defined as decisions based on “other-regarding preferences that are conditional on the perceived intentions behind the actions of others” [39]. Although expectations about reciprocity may facilitate the emergence of trust, trust can exist in the absence of reciprocity [48]. In our model, trust is based on the expectation that the agent will not comply less when the audit probability is lower. In other words, positive expectations do not refer to other-regarding preferences that ultimately benefit the regulator (e.g., higher compliance as a response to positive reciprocity). Instead, positive expectations refer to *self-regarding behavior that does not harm the regulator*. From a mathematical perspective, positive expectations in this model refer to actions that lead to non-negative outcomes. Reciprocity may further increase the benefits of trust-based regulation [17]. Explicitly incorporating the effects of reciprocity may be an interesting extension for future research.

Recently, experimental researchers have examined the effects of conditional audit strategies on compliance behavior [49, 50]. New experiments may test this model’s predictions by examining, for example, whether a reduction in the audit probability affects compliance, while controlling for long-term orientation. The second direction relates to the model that we employ, which has the same limitations as the generic game theoretical model of tax compliance [11]. For instance, one limitation is that behavior is dichotomous (i.e., the agent either fully pays taxes or pays nothing at all). Future models of trust-based regulation may incorporate more complex extensions, such as under-reporting, moral behavior, or social stigma, as compliance can be explained by economic and non-economic factors [28].

## Supporting Information

S1 Appendix Proofs. (PDF)
