# Supplementary material for: Only the Carrot, Not the Stick: Incorporating Trust into the Enforcement of Regulation
Source: PLoS One. 2015 Feb 23;10(2):e0117212. doi: 10.1371/journal.pone.0117212 (PMC4338102; doi:10.1371/journal.pone.0117212)
Supplement: S1 Appendix — (PDF) [file pone.0117212.s001.pdf]

Proof of Proposition 1

The value $\rho_d = \frac{\tau}{\tau+z}$ follows immediately from equating $VA_d|\{\alpha_d = 0\}$ and $VA_d|\{\alpha_d = 1\}$, where VA_d is given by (1). Similarly, (4) is derived.

Because the agent is made indifferent between complying and not complying, VA_k follows after evaluating these functions in $\alpha_k = 1$ and solving for VA_k for each $k \in \{w,\, d,\, t\}$.

Substituting $VA_d = \frac{1-\tau-\rho_d c}{1-\delta_A}$, $VA_t = \frac{1-\tau-\rho_t c}{1-\delta_A}$, and $\rho_d = \frac{\tau}{\tau+z}$ into (4) and solving for ρ_t yields two solutions: $\rho_t = \frac{\tau}{\tau+z}$ and $\rho_t = \frac{\tau+z}{c}\frac{1-\delta_A}{\delta_A}$. Note that the solution $\rho_t = \frac{\tau+z}{c}\frac{1-\delta_A}{\delta_A}$ is decreasing in δ_A. Solving $\frac{\tau+z}{c}\frac{1-\delta_A}{\delta_A} - \frac{\tau}{\tau+z} = 0$ yields $\delta^\star$.

The agent's strategy as formulated in (6)-(8) follows from equating $VR_k|\{\rho_k = 0\}$ and $VR_k|\{\rho_k = 1\}$, where VR_k is given by (2). Because the regulator is made indifferent between auditing and not auditing, VR_k follows after evaluating these functions in $\rho_k = 0$ and solving for VR_k.

Now suppose that $\alpha_d > \alpha_t$. Then $VR_d > VR_t$, which however would imply from (6) and (7) that $\alpha_d < \alpha_t$. Similarly, a contradiction is found for the alternative assumption $\alpha_d < \alpha_t$. Therefore, the only solution is $\alpha_d = \alpha_t$. The proof for α_w is similar. Indeed, when solving for α_t in the way we did for ρ_t, we again find two solutions: $\alpha_t = \alpha_d$ and $\alpha_t = \frac{\tau+z-\delta_R z}{\delta_R \tau} > 1$. This second solution is therefore not feasible.

Proof of Proposition 2

Assume that the discount factor δ_A is drawn from some distribution function, the regulator learns the agent's δ_A after the first audit, and sets the audit probability based on the following three conditions:

A. If the agent complies and $\delta_A < \delta^\star$, then the agent faces ρ_d in the next period.

B. If the agent complies and $\delta_A > \delta^\star$, then the agent faces ρ_t in the next period.

C. If the agent does not comply, the agent faces ρ_d in the next period.

After the first audit, the regulator knows the agent's discount factor, so the equilibria of Proposition 1 apply. Because $\rho_t = \frac{\tau}{\tau+z+\delta_A(VA_t-VA_d)}$, learning that $\delta_A < \delta^\star$ would make the regulator *increase* the audit probability in the trusted state (i.e., $\rho_t > \rho_d$), but this contradicts the principle of trust. Proposition 1 shows that the regulator is indifferent between the games with and without trust. The regulator's payoffs are not affected by auditing an agent with $\delta_A < \delta^\star$ with probability ρ_d instead of ρ_t, so the regulator can credibly commit to applying condition A.

Now define $\tilde{\rho}$ as the audit probability that makes an agent with $\delta_A = \tilde{\delta}$ indifferent between complying and not complying. Given $\tilde{\rho}$, the agent's pure strategy is to comply if $\delta_A > \tilde{\delta}$, and not to comply when

$\delta_A < \tilde{\delta}$. Only when $\tilde{\rho} = \rho_d$, an agent with $\delta_A < \tilde{\delta}$ is indifferent between complying and not complying.

In the waiting state, the value function of the regulator is given by

$$VR_w = \alpha\tau + \rho_w[(1-\alpha)(\tau + z - \delta_R(VR_t - VR_d)) - s + \delta_R VR_t] + (1-\rho_w)VR_w,$$

After the first audit, the equilibria of Proposition 1 apply, so $VR_t = VR_d$, and the value function simplifies to

$$VR_w = \frac{\alpha\tau + \rho_w[(1-\alpha)(\tau+z) - s + \delta_R VR_t]}{1-\delta_R(1-\rho_w)},$$

where α is a function of ρ as explained above.

Taking the derivative with respect to ρ yields:

$$sign\left\{\frac{\partial VR_w}{\partial \rho}\right\} = sign\left\{[\tau + z(1-\delta_R)](1 - \frac{s}{\tau+z} - \alpha(\rho)) + (1-\delta_R+\delta_R\rho)[\tau - \rho(\tau+z)]\alpha'(\rho)\right\} \quad (1)$$

Now let us analyze the following two cases:

i. The regulator's assessment of δ_A is such that $\Pr\{\delta_A > \delta^\star\} > \alpha^\star$. Then, when $\rho \geq \rho_d = \frac{\tau}{\tau+z}$, the second term of the right-hand side of (11) is less than or equal to zero and the first term is negative. The latter holds because $\Pr\{\delta_A > \delta^\star\} > \alpha^\star$ implies that $\alpha(\rho) > 1 - \frac{s}{\tau+z}$. The derivative can thus only be equal to zero for $\rho < \rho_d$. However, if $\alpha(\rho) \leq 1 - \frac{s}{\tau+z}$, the first term is greater than or equal to zero, and this can only follow from $\rho < \rho_d$, which implies that the second term is also positive. Therefore VR is maximized for some $\rho < \rho_t$ that yields $\alpha(\rho) > \alpha^\star$.

ii. The regulator's assessment of δ_A is such that $\Pr\{\delta_A > \delta^\star\} \leq \alpha^\star$. When $\rho = \rho_d$, the second term of the right-hand side of (11) equals zero and the first term is positive unless an agent with $\delta_A < \delta^\star$ uses a mixed strategy. Now let $\rho_w = \rho_d$. When $\delta_A > \delta^\star$, the waiting agent has a pure strategy to comply. When $\delta_A \leq \delta^\star$, let the waiting agent comply with probability $\hat{\alpha} = \frac{\alpha^\star - \Pr\{\delta_A > \delta^\star\}}{\Pr\{\delta_A < \delta^\star\}}$. From the regulator's point of view, the compliance probability is equal to $\Pr\{\delta_A > \delta^\star\} \cdot 1 + \Pr\{\delta_A < \delta^\star\} \cdot \hat{\alpha} = 1 - \frac{s}{\tau+z}$, which indeed makes the regulator indifferent between auditing and not auditing (as shown in Proposition 1). Indeed, in such case $\frac{\partial VR_w}{\partial \rho} = 0$.

   For the agent, the optimal strategy is to comply when $\delta_A > \delta^\star$ because it results in entering the trust state, and to comply with probability $\hat{\alpha}$ when $\delta_A < \delta^\star$ because she is indifferent between

complying and not complying (in such case, $\rho_w = \rho_t = \rho_d$ and therefore $VA_t = VA_d$).

Finally, we note that when $\Pr\{\delta_A > \delta^\star\} = 0$, solution *ii* is equivalent to the deterrence-based equilibrium, so that a trust-based solution does not exist.

From the value functions of the regulator, which are given in Proposition 1, it follows that the regulator has the same expected payoffs with and without trust-based regulation.

Proof of Proposition 3

This proof goes along the same lines as the proof of Proposition 1. Let us start with the compliance probabilities. The value for the regulator is again given by $VR_k = \frac{\alpha_k \tau}{1-\delta_R}$. For the compliance probabilities α_k, α_j, with $k,\, j \in \{w,\, d,\, t\}$, it follows from these value functions ($VR_k = \frac{\alpha_k \tau}{1-\delta_R}$) that $\alpha_k > \alpha_j$ would imply that $VR_k > VR_j$. However, (10) would imply that $\alpha_k < \alpha_j$, so the only possible solution is $\alpha_w = \alpha_d = \alpha_t$. The value $1 - \frac{s}{\tau+z}$ follows from the fact that the term $VR_k - VR_j \,\forall\, k,\, j \in \{w,\, d,\, t\}$ equals zero.

The values for ρ_t and ρ_d are obtained by solving the system of equations given by (10), while expressing the values using α_k and solving for VA_k. Now, solving VA_d for $\alpha_d = 0$ and VA_t for $\alpha_t = 1$ yields:

$$VA_d = \frac{1 - \rho_d(\tau + z + c)}{1 - \delta_A} \text{ and } VA_t = \frac{1 - \tau - \rho_t c}{1 - \delta_A}.$$

Substituting these expressions in (10) yields the following two equations:

$$\begin{aligned} \rho_d &= \tfrac{\tau \rho_t}{u\tau + \rho_t(\tau+z)(1-u)} \\ \rho_d &= \tfrac{\tau + \rho_t c}{\tau + z + c} + \tfrac{(1-\delta_A)\tau - \rho_t(\tau+z)}{\delta_A \rho_t(\tau+z+c)} \end{aligned}$$

Solving for ρ_t yields the two solutions given in the proposition. The third root is

$$\frac{1}{2c\delta_A(1-u)}\left[F - \sqrt{F^2 + 4c\delta_A(1-u)\tau u(1-\delta_A)}\right],$$

which is not a feasible solution as this root is strictly negative.

Finally, it can be verified for this solution that $\rho_t = \rho_d$ if and only if

$$u = \frac{c\delta_A \tau - (1-\delta_A)(\tau+z)^2}{\delta_A \tau(\tau + z + c)}.$$

Because $u^\star = 0$ for $\delta_A = \delta^\star$, $u^\star$ is strictly increasing in δ_A, and $u^\star = \frac{\tau c}{\tau(c+\tau+z)} < 1$ for $\delta_A = 1$, it follows that $u^\star \in (0,1)$ so that the trust-based solution satisfies $\rho_d > \rho_t$ iff $u < u^\star$. This completes the proof.
